# Improved cryopreservation of cardiomyocyte aggregates differentiated from GMP iPSC in a 3D culture format

**DOI:** 10.1038/s41598-025-32439-3

**Published:** 2026-01-12

**Authors:** Fabienne Becker, Soraia Martins, Carlos A. Hernandez-Bautista, Boris Greber, Robert Zweigerdt, Gesine Kogler

**Affiliations:** 1 Institute for Transplantation Diagnostics and Cell Therapeutics, Medical Faculty, Heinrich-Heine-University, Düsseldorf, Germany; 2 Catalent Düsseldorf GmbH, Langenfeld, Germany; 3 Department of Cardiothoracic, Transplantation and Vascular Surgery (HTTG), Leibniz Research Laboratories for Biotechnology and Artificial Organs (LEBAO), REBIRTH-Research for Translational Regenerative Medicine, Hannover Medical School, Hannover, Germany

**Keywords:** Biological techniques, Biotechnology, Cardiology, Cell biology, Stem cells

## Abstract

Human induced pluripotent stem cell-derived cardiomyocytes (hiPSC-CMs) are in the focus of clinical research for cell-based therapies for heart failure patients. However, challenges include long-term storage of cardiomyocyte aggregates (CMAs). Current freeze/thawing protocols have been established for single cells, indicating a substantial gap for process development and optimization for cryopreservation. Here CMA differentiation was achieved by the combination of WNT-pathway modulation and TGF-β/SMAD- and FGF-pathway targeting. An efficient CMA differentiation with cardiac marker expression of ≥ 95% ACTN2/TNNT2 was established. For process optimization, different freezing media were combined with a pre-treatment strategy and comprehensive assessment of cell recovery, viability, physiology, and purity was performed before and after cryopreservation. Development and optimization of cryopreservation strategies for CMAs led to a freezing medium termed “10% human serum albumin (HSA)” representing the best option tested. Analysis at 5-days post thawing revealed maintenance of high cardiac marker expression (> 90%), spontaneous contraction activity, and overall recovery of > 80% vital cell counts compared to sample analysis before the freezing procedure. Extensive modification and optimization of established single cell CM cryopreservation protocols was achieved with successful recovery of CMs within the complex structure of aggregates.

**Supplementary Information:**

The online version contains supplementary material available at 10.1038/s41598-025-32439-3.

## Introduction

Cardiovascular diseases are a prevalent problem in modern medicine due to the high mortality rates resulting from the limited regenerative capacity of the heart^1^. However, progress has been made in recent years not only in prolonging patients’ life, but also in significantly increasing their quality of life, utilizing different approaches in the fields of organ transplantation, artificial heart devices or drug treatments^2,3^. Additionally, novel approaches in the field of cell-based therapies have emerged as another option for the treatment of heart diseases. In recent years, there has been a shift in clinical trial strategies toward exploring the therapeutic potential of cardiac cells generated from human pluripotent stem cells (hPSCs), e.g., for the treatment of ischemia^4,5^. For instance, Zimmermann et al. have demonstrated the feasibility of utilizing engineered heart muscle allografts for the remuscularization of the heart, leading to the approval of their in-human clinical trial on tissue-engineered heart repair^6^. Various differentiation protocols have been established for the production of cardiomyocytes from both embryonic stem cells (ESCs) and induced pluripotent stem cells (iPSCs)^7,8^. The accumulation of different optimization approaches has led to the robust production of highly pure cardiomyocytes (CMs) and other cardiac cell types, which are in turn readily available for application in both research and clinical settings. In recent years there has been a focus on novel techniques for the scalable production of cardiomyocytes – differentiation in 3D-based models. The advantage of utilizing e.g., 3D aggregates, engineered tissues, or cardioids, is their ability to partially recapitulate cardiac physiology, while enabling reproducible high throughput applicability and being both affordable and technically simple^9^. Due to the cell–cell connectivity, these new 3D-based cardiac models can potentially be applied for tissue engineering, drug development, and for cardio toxicology screening and disease modeling^10^. Many optimized differentiation protocols allow for the direct production of cardiomyocytes in small-scale well-plate culture systems or on a larger scale via the utilization of spinner flasks or bioreactors^11–14^.

A remaining bottleneck for the translation of cell-based therapies (both single cell or 3D-based models) toward good manufacturing practice (GMP) compliant production for possible clinical use, is the preparation time and subsequent cell/aggregate storage mandatory for straightforward downstream logistics, including cell product transportation and application at the bedside^15,16^. Cryopreservation allows for long-term storage and subsequent distribution of the cell product, thereby uncoupling cell production from the application and allowing for the regulatory safety and sterility testing required before therapeutic use. Several studies have shown sufficient cryopreservation protocols with high viability directly post-thaw for single cell cardiomyocytes. Most commonly CMs are frozen in either commercially available freezing media (e.g. Cryostor CS-10 or Stemdiff Cardiomyocyte Freezing Medium), or KnockOut Serum Replacement (KOSR) or Fetal Bovine Serum (FBS) with the addition of DMSO as the cryoprotectant^17–23^. While commercially available freezing media are chemically defined and include additional stabilizers e.g., sugars, antioxidants, and ionic buffers, to enhance the cryoprotection by protecting cell–cell junctions and limiting osmotic shock^24,25^, most are not manufactured GMP-grade. Therefore, it is common practice to utilize culture medium supplemented with DMSO as a cryoprotectant. DMSO is a cell membrane permeable protectant that reduces oxidative stress and inhibits intracellular ice nucleation via dehydration of the cell^26,27^. However, a limitation of DMSO is the cytotoxicity, due to which the right handling of the cells once the cryoprotectant is added is crucial for preventing cell death^28^. The cryopreservation of cardiomyocyte spheroids or organoids is less well characterized. Due to the complex structure and function of spheroids/organoids, existing protocols need to be optimized to accommodate different freezing properties, e.g., ensuring effective nutrient and cryoprotectant transport into the tissue. Initial efforts have been made to achieve efficient cryopreservation of both small cardiac spheroids and more complex engineered cardiac tissue^29,30^.

For the utilization of cryopreservation as an intermediate long-term storage option for hPSC-derived CMs between culture and possible clinical application, standardized quality assessment and quality control (QA/QC) analyzes should be implemented^31,32^. Moreover, most studies assess cardiomyocyte (CM) viability immediately after thawing, which may lead to an overestimation of cryopreservation efficiency, as apoptosis induced by cryoinjury often becomes apparent only after a delayed time frame^33^. As cryopreservation potentially causes cell damage, which could irreversibly change the quality of the cells, i.e. reduce viability, cause phenotypic changes, or impact function, possible QA/QC assays need to be tailored toward different assessment methods to obtain a broad overview of the quality of cryopreserved cells without neglecting crucial parameters needed for possible clinical application, as well as taking recovery time into account.

The aim of this study was to evaluate different cryopreservation media and Y-27632 pre-treatment suggested for the conventional cryopreservation of single cell suspensions, to identify the most suitable compounds and strategies for the successful freezing of more complex 3D cardiomyocyte aggregates. Moreover, this study proposes more specific and comprehensive assessment methods for the characterization of CMs after cryopreservation to provide more relevant and reliable assessments of freezing process modulations.

## Results

### Efficient differentiation of cardiomyocyte-aggregates from a HLAh hiPSC line in different 3D-suspension culture systems

**Fig. 1:**
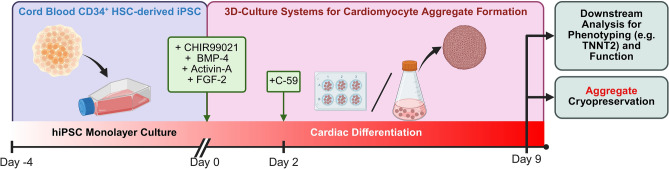
Schematic of the differentiation protocol. Utilizing cord blood CD34^+^ HSC-derived iPSC for the differentiation into cardiomyocyte-aggregates in a 3D-suspension culture. Created in https://BioRender.com.

The HLAh iPSC line R26 was differentiated towards cardiomyocytes both in small-scaled 6-well plates (6WP) (working volume 2 mL to 4 mL), as well as larger-scaled 125 mL Erlenmeyer flasks (EFlask) (working volume 20 mL) in 3D-suspension culture (schematic in Fig. 1).

**Fig. 2:**
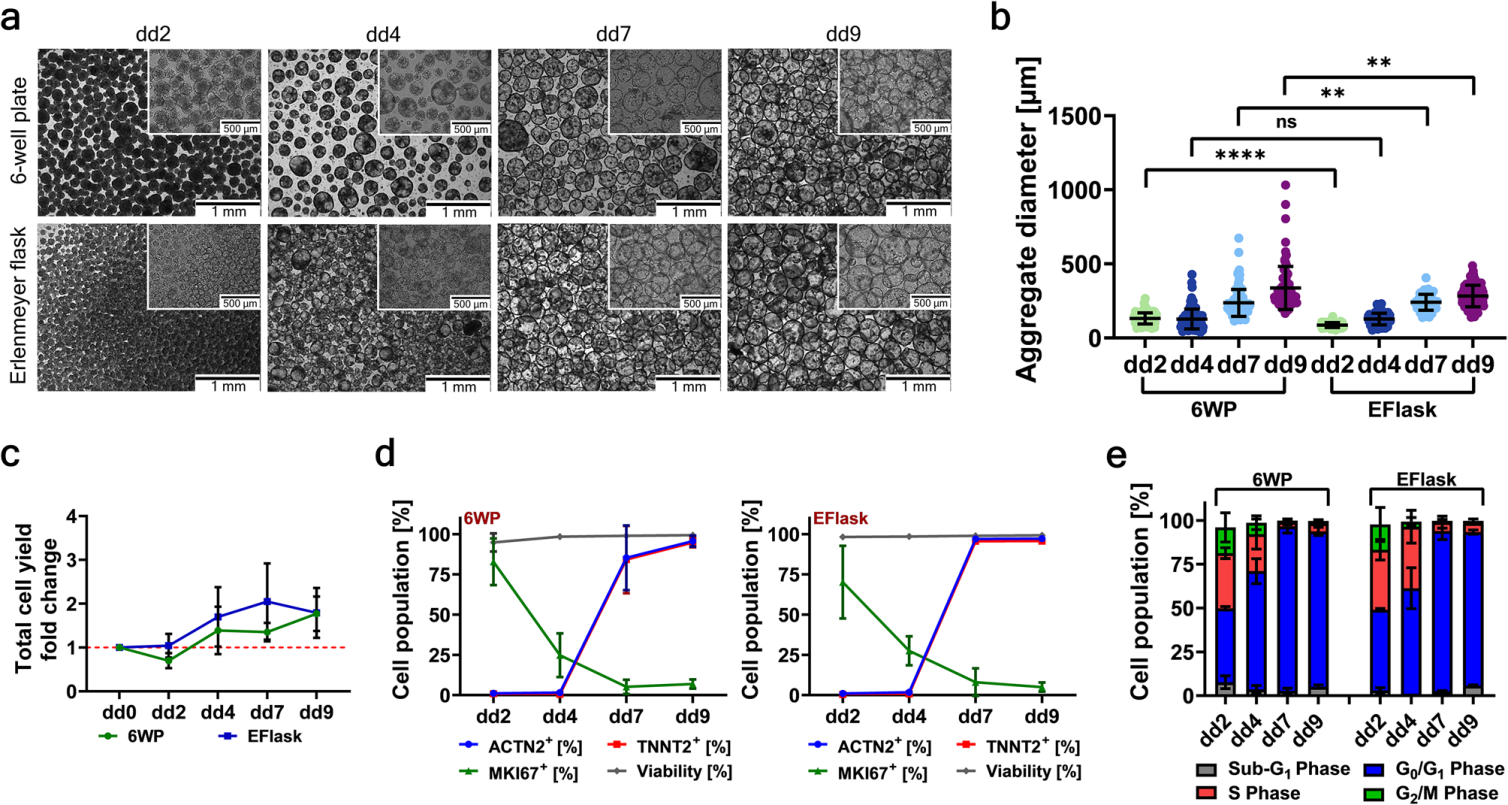
3D-suspension culture cardiomyocyte differentiation characteristics. (**a**) Representative bright field images for morphological assessment at different time-points of differentiation. Scale bar, 1 mm and 500 µm. (**b**) Aggregate diameter distribution during differentiation; shown are individual values of aggregates from n = 3 experiments and mean values ± SD (for each experiment > 40 aggregates were measured); Data were analyzed for statistical significance using an unpaired t-test (**p ≤ 0.01, ****p ≤ 0.0001). (**c**) Cell yields depicted as fold changes at different time-points during differentiation (mean values ± SD, n = 3). (d) Flow cytometry analysis for cardiac markers ACTN2 and TNNT2, proliferation marker MKI67, and viability (mean values ± SD, n = 3). (**e**) Cell cycle phase distribution during differentiation (mean values ± SD, n = 3).

Dissociated, single iPS cells from a 2D-monolayer culture were transferred into 3D-suspension culture onto a shaker for promoting their self-assembly into aggregates (~ 130 µm average diameter for 6WP, and ~ 90 µm average diameter for EFlask) in the span of < 2 days (Fig. 2a). During the 9-day differentiation process, the aggregates steadily grew, reaching a 2–3 times increase from their initial aggregate size. Depending on the platform used for the differentiation (6WP or EFlask), the CMA size differs significantly (Fig. 2b). CMAs generated in 6WP reached an average sizes of about 337 µm ± 144 µm. CMAs generated in EFlasks reached significantly smaller and more homogeneous average aggregate sizes of about 283 µm ± 72 µm. Both platforms showed a ~ 1.8-fold increase in cell yield compared to the seeded number of iPS cells (Fig. 2c). Cell suspensions derived from differentiation day (dd) 9 CMAs by dissociation showed high cardiac marker expression for both ACTN2 (95.6% ± 3.4% and 97.2% ± 0.6%, for 6WP and EFlask differentiation respectively) and TNNT2 (94.8% ± 3.0% and 95.6% ± 1.1%, for 6WP and EFlask differentiation respectively) and high cell viability (≥ 99%) independent of the platform used for CMA production (Fig. 2d).

Cell proliferation analysis on dd9 revealed relatively low MKI67 expression (≤ 7%) and a high percentage of cells that were predominantly in the G_0_/G_1_ phase (≥ 87%) of the cell cycle and only a low percentage of cells in either S (≤ 5%) or G_2_/M (≤ 2%) phase (Fig. 2d-e). The potential presence of residual iPSC contamination was assessed by flow cytometry analysis of the pluripotency marker POU5F1 and by analyzing the gene expression level of miR-302/367 HT (Extended Data Fig. 1). iPSC-spiking of various concentrations (50% to 0.01%) indicated the detection of ~ 0.1% residual iPSC in a sample via flow cytometry and the sensitive detection of as low as 0.01% residual iPSC in a sample via real-time qPCR. Analyzing CMAs during the differentiation process revealed no residual iPSC contamination by dd9 (< 0.1% POU5F1 expression measured via flow cytometry and < -1000-fold down regulated gene expression of miR-302/367 HT measured via qPCR). Additionally, a selection of CD-markers were utilized to further define the TNNT2^-^ population present after dd9 (Extended Data Fig. 2d). The characteristic marker for endothelial cells (CD31^+^), hematopoietic cells (CD45^+^ and CD34^+^) and for fibroblasts (CD90^+^, CD73^+^ and CD105^+^) were negative.

### Efficient cryopreservation of cardiomyocyte-aggregates through the addition of human serum albumin into the freezing medium

Due to the complex structure of cardiac aggregates, cryopreservation effects were assessed to determine the influence of freezing on structure, viability, phenotype, and function. CMAs from dd9 onward (dd9 to dd11) expressing > 85% ACTN2 and TNNT2 were further utilized for cryopreservation purposes. Because size was the only discriminating factor in CMAs generated in either 6WP or EFlask (Fig. 2b 337 µm ± 144 µm and 283 µm ± 72 µm respectively; **p ≤ 0.01), the results for their cryopreservation were summarized independent of the platform used for the differentiation.

Both commercially available freezing media (Stemdiff-CMFM, CS-10, Nutrifreeze-D10, and Stem Cellbanker) and basic freezing media with different additives (CMFreeze, 10% HSA, 10% KOSR, 90% KOSR) were tested for their cryoprotective capabilities for the cryopreservation of CMAs.

**Fig. 3:**
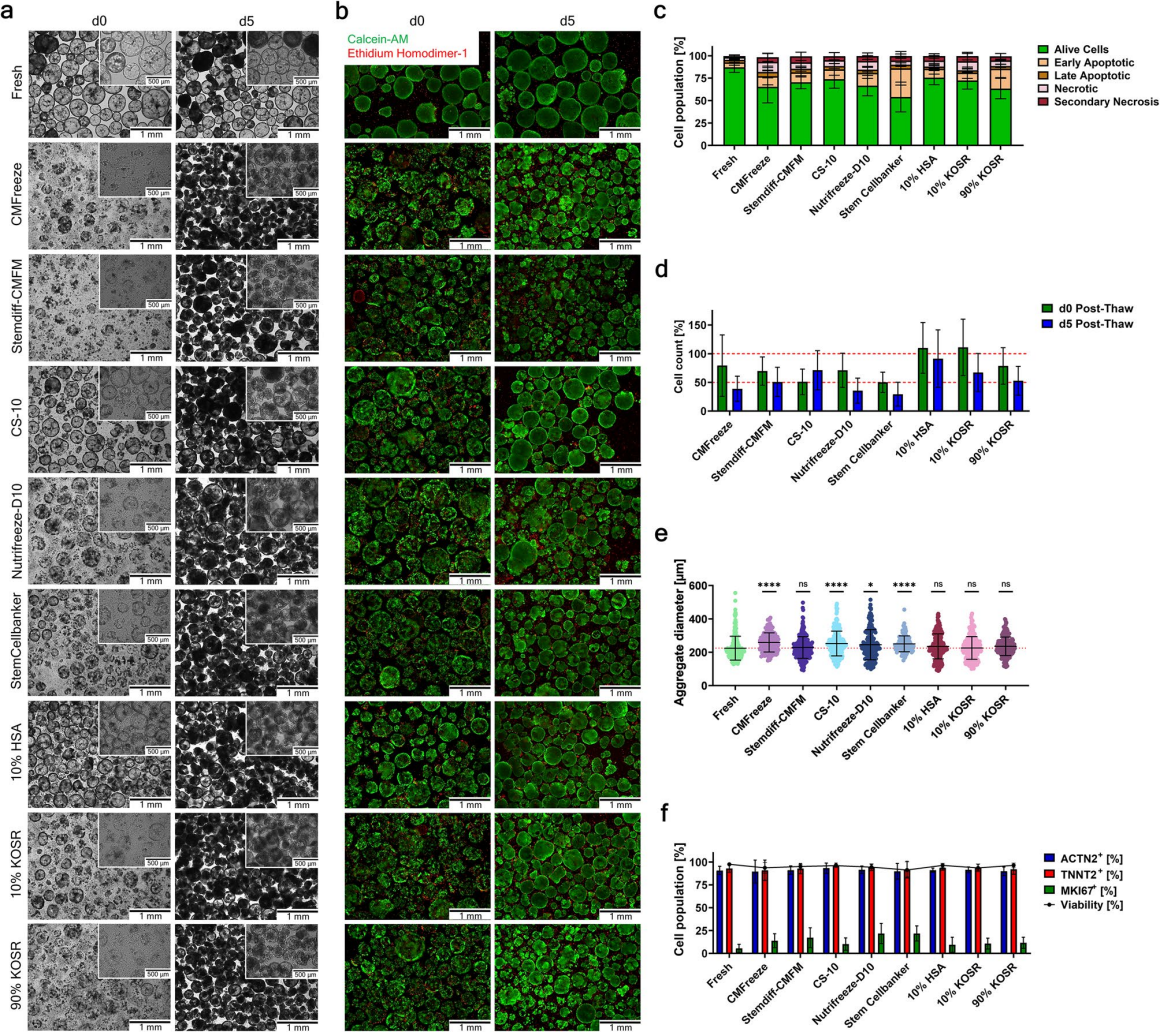
Cryopreservation of cardiomyocyte-aggregates. Comparison of different freezing media for the cryopreservation of CMAs. (**a**) Representative bright field images for morphological assessment of CMA integrity d0 and d5 post-thaw. Scale bar 1 mm and 500 µm. (**b**) Representative fluorescent images of CMAs d0 and d5 post-thaw for viability assessment. Staining with Calcein-AM (green) and Ethidium homodimer-1 (red). Scale bar 1 mm. (**c**) Apoptosis assay via annexin V / PI on d0 post-thaw. (d) Cell recovery analyzed immediately on d0 post-thaw and after a 5-day post-thaw culture (n = 5–8, mean values ± SD, the red dotted line marks both 50% and 100% recovery)). (e) Aggregate diameter distribution on d5 post-thaw; shown are individual values of aggregates from n = 5–8 experiments and mean values ± SD (for each experiment > 30 aggregates were measured, the red dotted line indicated the mean value of the fresh sample); Data were analyzed for statistical significance using an unpaired t-test (*p ≤ 0.05, ****p ≤ 0. 0001). (**f**) Flow cytometry analysis for cardiac markers ACTN2 and TNNT2, proliferation marker MKI67, and viability on d5 post-thaw (mean values ± SD, n = 5–8). In all analyses the condition “Fresh” indicated CMAs analyzed prior to freezing as an age matched alive control; n = 5 referred to the conditions CMFreeze, Nutrifreeze-D10, and Stem Cellbanker; n = 8 referred to Stemdiff-CMFM, CS-10, 10% HSA, 10% KOSR, and 90% KOSR.

On d0 post-thaw, morphology assessment of cryopreserved CMAs revealed a heavily impaired aggregate structure with most aggregates displaying a disrupted outer membrane, independent of the freezing medium (Fig. 3a). Freezing media CS-10 and 10% HSA demonstrated superior protection of the aggregate structures, with a minimal disruption of CMA integrity. On the other hand, the freezing media CMFreeze, Stemdiff-CMFM, and 90% KOSR had the most drastic CMA structure disruption immediately post-thaw. However, after a 5-day post-thaw culture, all conditions were able to recover the aggregate integrity of most CMAs. Compared to the fresh sample, CMAs that underwent freezing appeared to have a denser structure compared to the more cystic appearance of the fresh cells.

Even though aggregate structure was compromised immediately after cryopreservation, whole aggregate viability staining revealed that most of the cells in the aggregates were alive and only a few cells in the outer regions of the aggregates were dead as well as single cells that had detached from the aggregates (Fig. 3b and Extended Data Fig. 2). After the post-thaw culture (d5), the CMAs were completely viable and the only dead cells in the culture were the remaining single cells that had detached from the aggregates. Further assessment of the viability of CMAs directly post-thaw through flow cytometry analysis with an apoptosis assay (Annexin V/PI) revealed first signs of ongoing apoptosis of cells in all conditions (Fig. 3c). To ensure the detected apoptotic and necrotic cells were a direct result of the cryopreservation process and not due to the dissociation of the CMAs, as the process can potentially result in specific membrane damage and therefore lower levels of viable cells, a sample of fresh CMAs was included. After dissociation of fresh CMAs, 87.2% ± 5.7% of cells were alive, while 5.4% ± 3.0% and 3.0% ± 0.8% were early and late stage apoptotic, and 3.0% ± 2.5% and 1.2% ± 0.7% were necrotic or secondary necrotic. As evidenced, the dissociation process has a slight effect on the viability of CMs and initiated low levels of apoptosis/necrosis. After cryopreservation, all conditions had a lower number of viable cells (≤ 75%) and a higher number of cells of both early stages of apoptosis (9%-32%) and necrosis (5%-11%). Both late stage apoptotic and secondary necrotic cell levels were similar to those of fresh cells (3%-4% and 1%-7% respectively). CMAs frozen with Stem Cellbanker had the lowest number of alive cells (53.9% ± 16.7%), while CMAs cryopreserved with either Stemdiff-CMFM, CS-10, 10% HSA, or 10% KOSR had the highest living cell numbers (70.4% ± 7.2%, 73.9% ± 10.1%, 75.6% ± 7.9%, and 72.4% ± 9.6% respectively).

To evaluate the impact of post-thaw cryoinjury, cell recovery was assessed both immediately after thawing (d0) and following a short post-thaw culture period (d5) (Fig. 3d). Immediately post-thaw (d0), most conditions had high recoveries of ≥ 50%, with both the conditions 10% HSA and 10% KOSR performing best out of the tested freezing media with recoveries of ~ 110%. By d5, most conditions had a severe drop in the recovery. The commercially available freezing medium Stem Cellbanker had the most profound cell loss with a recovery of only 29.2% ± 20.8%, followed by Nutrifreeze-D10 with 35.5% ± 21.9%, and CMFreeze with 38.8% ± 21.9%. Moderate recoveries were achieved with either Stemdiff-CMFM (50.7% ± 25.6%), or 90% KOSR (52.7% ± 25.1%). The best results were accomplished with either 10% KOSR (67.3% ± 33.2%), CS-10 (71.2% ± 34.2%), or 10% HSA (91.2% ± 50.1%). Notably, all conditions had a high batch-to-batch variability as evidenced by the high SD values. In addition to the cell recovery, mean aggregate diameters were analyzed to rule out a size bias of the cryopreservation protective capacities towards different sized CMAs (Fig. 3e). Compared to the CMA size distribution of the fresh sample (225 µm ± 71 µm), many conditions (Stemdiff-CMFM, 10% HSA, 10% KOSR, and 90% KOSR) had no significant change in the CMA size on d5 post-thaw. CMAs cryopreserved with Nutrifreeze-D10 had a small increase in the mean diameter and overall size distribution (245 µm ± 90 µm, *p ≤ 0.05). On the other hand, CMAs cryopreserved with either CMFreeze, CS-10, or Stem Cellbanker had a higher difference in their size distribution (significance of ****p ≤ 0.0001) with mean diameters reaching sizes of 259 µm ± 57 µm, 256 µm ± 76 µm, and 250 µm ± 48 µm respectively.

The recovered CMAs were characterized via flow cytometry for their cardiac marker (ACTN2 and TNNT2) and proliferation marker (MKI67) expression and their viability on d5 post-thaw (Fig. 3f). All conditions displayed comparable levels of ACTN2 and TNNT2 expression (~ 90% and ~ 93% respectively). The proliferation capacity of the CMAs was slightly elevated in all conditions as evidenced by higher MKi67 expression (≥ 10%) as compared to the fresh sample (5.46% ± 4.39%). Similar to the observed high viability of the whole aggregates (Fig. 3b), flow cytometry analysis confirmed that by d5 post-thaw the CMAs were highly viable (≥ 91%) independent of the freezing media.

As a proof of principal, dissociated CMAs have been frozen in the best working cryoprotectant “10% HSA”. Upon thawing of the single cardiomyocytes initial recovery was ~ 94%, but after the 5-day post-thaw monolayer culture, only ~ 63% of plated cells were recovered (Extended Data Fig. 4).

### Effect of Y-27632 pre-incubation on cardiomyocyte-aggregates prior to cryopreservation

**Fig. 4:**
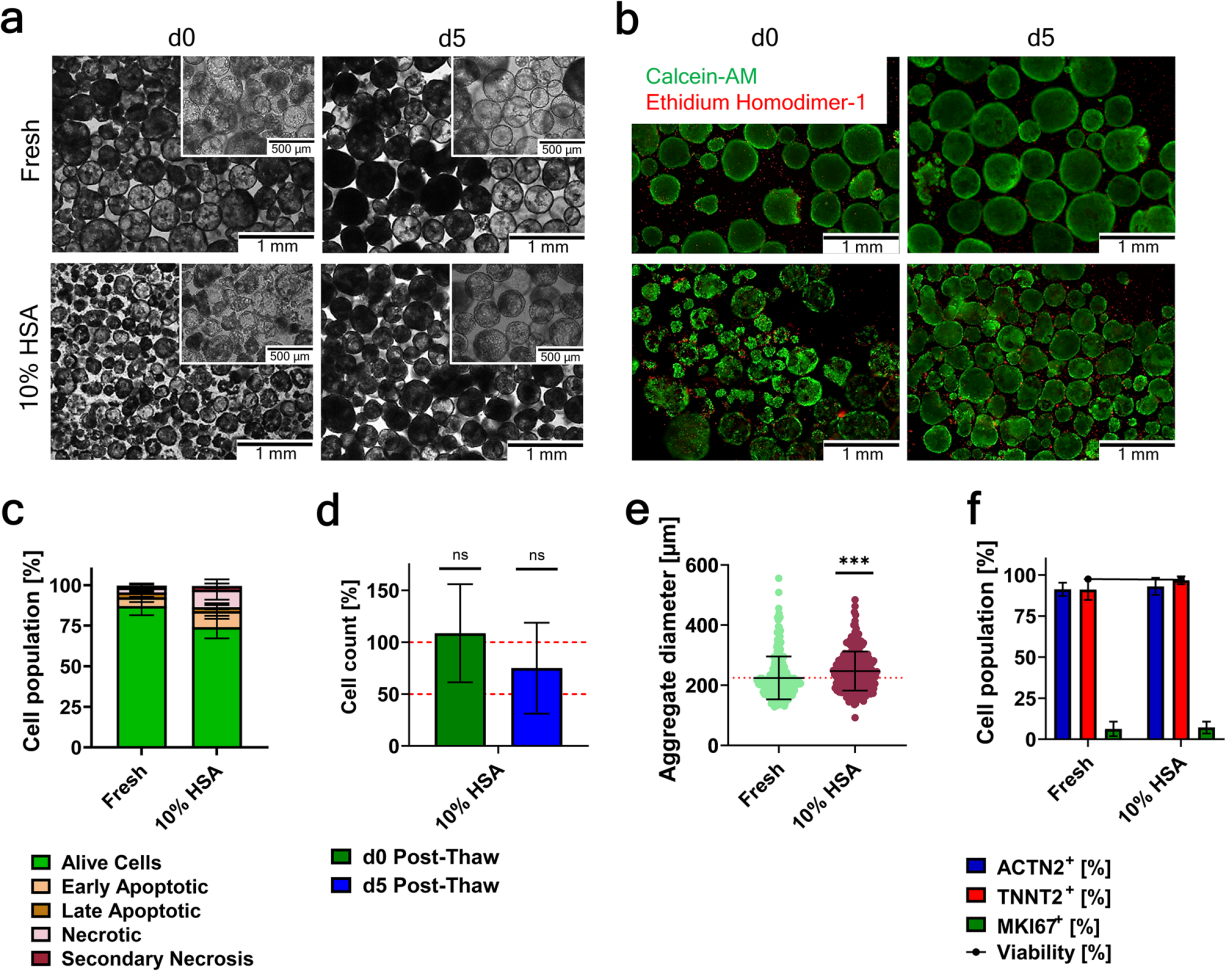
Cryopreservation of cardiomyocyte-aggregates pre-treated with Y-27632 in 10% HSA. (**a**) Representative bright field images for morphological assessment of CMA integrity d0 and d5 post-thaw. Scale bar 1 mm and 500 µm. (**b**) Representative fluorescent images of CMAs d0 and d5 post-thaw for viability assessment. Staining with Calcein-AM (green) and Ethidium homodimer-1 (red). Scale bar 1 mm. (**c**) Apoptosis assay via annexin V / PI on d0 post-thaw. (**d**) Cell recovery analyzed immediately on d0 post-thaw and after a 5-day post-thaw culture (n = 8, mean values ± SD, the red dotted line marks both 50% and 100% recovery)). (**e**) Aggregate diameter distribution on d5 post-thaw; shown are individual values of aggregates from n = 8 experiments and mean values ± SD (for each experiment > 30 aggregates were measured; the red dotted line indicated the mean value of the fresh sample); Data were analyzed for statistical significance using an unpaired t-test (***p ≤ 0.001; in d data was compared to the corresponding data of CMAs cryopreserved without pre-treatment; in e data was compared to the fresh sample). (**f**) Flow cytometry analysis for cardiac markers ACTN2 and TNNT2, proliferation marker MKI67, and viability on d5 post-thaw (mean values ± SD, n = 8). In all analyses the condition “Fresh” indicated CMAs analyzed prior to freezing as an age matched alive control.

One optimization approach for the cryopreservation of CMAs was the 1 h pre-treatment of cells at 37 °C prior to cryopreservation with the Rho-associated kinase inhibitor Y-27632. As the condition “10% HSA” has been the best performing cryoprotectant for CMA freezing, the data is shown for this condition only (Fig. 4).

The pre-treatment of CMAs with 10 µM Y-27632 prior to cryopreservation had an immediate effect on the morphology of the thawed aggregates (Fig. 4a). On d0 post-thaw aggregate morphology appeared to be more intact, although closer inspection of the membrane integrity revealed some disruption in the aggregate structure. By d5 post-thaw, most aggregates had nearly completly recovered their structure.

Whole aggregate viability assessment revealed only marginal improvement of the CMA viability with Y-27632 pre-treatment on d0 post-thaw (Fig. 4b and Extended Data Fig. 6). As mentioned above, aggregates were made up of mostly viable cells, with dead cells predominantly in the outer regions of the aggregate or as detached single cells in the culture. By d5 post-thaw, CMAs were completely viable, with remaining dead cells only present as detached single cells in the culture medium. Further assessment of the viability of CMAs post-thaw with the apoptosis assay revealed no significant improvement for the number of alive cells (Fig. 4c). CMAs cryopreserved with 10% HSA had the highest number of alive cells (74.1% ± 7.0%) and lowest number of cells undergoing early apoptosis (9.9% ± 4.8%), similar as CMAs frozen with the medium but without the pre-treatment. There was no significant increase in cells in either late apoptosis or secondary necrosis as compared to both fresh cells and CMAs cryopreserved without pre-treatment.

To determine if pre-treatment of CMAs with Y-27632 prior to cryopreservation was able to inhibit onsetting cryoinjury effects post-thaw, cell recovery was calculated directly post-thaw (d0) and after a short post-thaw culture (d5) (Fig. 4d). Both the cell recovery immediately post-thaw (108.6% ± 47.2%) and after the 5-day culture (75.0% ± 43.8%) were not significantly increased in the pre-treated condition as compared to the CMAs cryopreserved without the Y-27632. In addition to the reduced recovery of CMAs after the post-thaw culture, aggregate size measurements revealed a significantly different size distributions by d5, with mean diameters of 247 µm ± 65 µm (Fig. 4e).

Analysis of cardiac markers revealed no significant difference in ACTN2 (≥ 91%) and TNNT2 (≥ 92%) levels as compared to the fresh sample (91.2% ± 4.0% and 91.2% ± 6.3% respectively). MKI67 expression levels (6.3% ± 4.4% for fresh CMAs and 9.0% ± 6.1% for cryopreserved CMAs) were similar (Fig. 4f). Albeit there was a reduced cell recovery, flow cytometry measurement of the viability of cells revealed high viability on d5 post-thaw (≥ 92%), which was in line with the fluorescence viability staining of the whole aggregates.

All previously tested freezing media were also used for cryopreservation with a Y-27632 pre-treatment (Extended Data Fig. 5). There was no significant increase in the overall cryoprotectant capacity.

### Recovery of the spontaneous contraction of cryopreserved cardiomyocyte aggregates

In addition to the confirmation of an unaffected phenotype and a high viability of CMAs after cryopreservation, their functional capacity was analyzed via a video-based assay of the aggregate’s spontaneous contraction. Here only CMAs cryopreserved with the best working freezing protocol (10% HSA ± 1 h Y-27632) were analyzed.

**Fig. 5:**
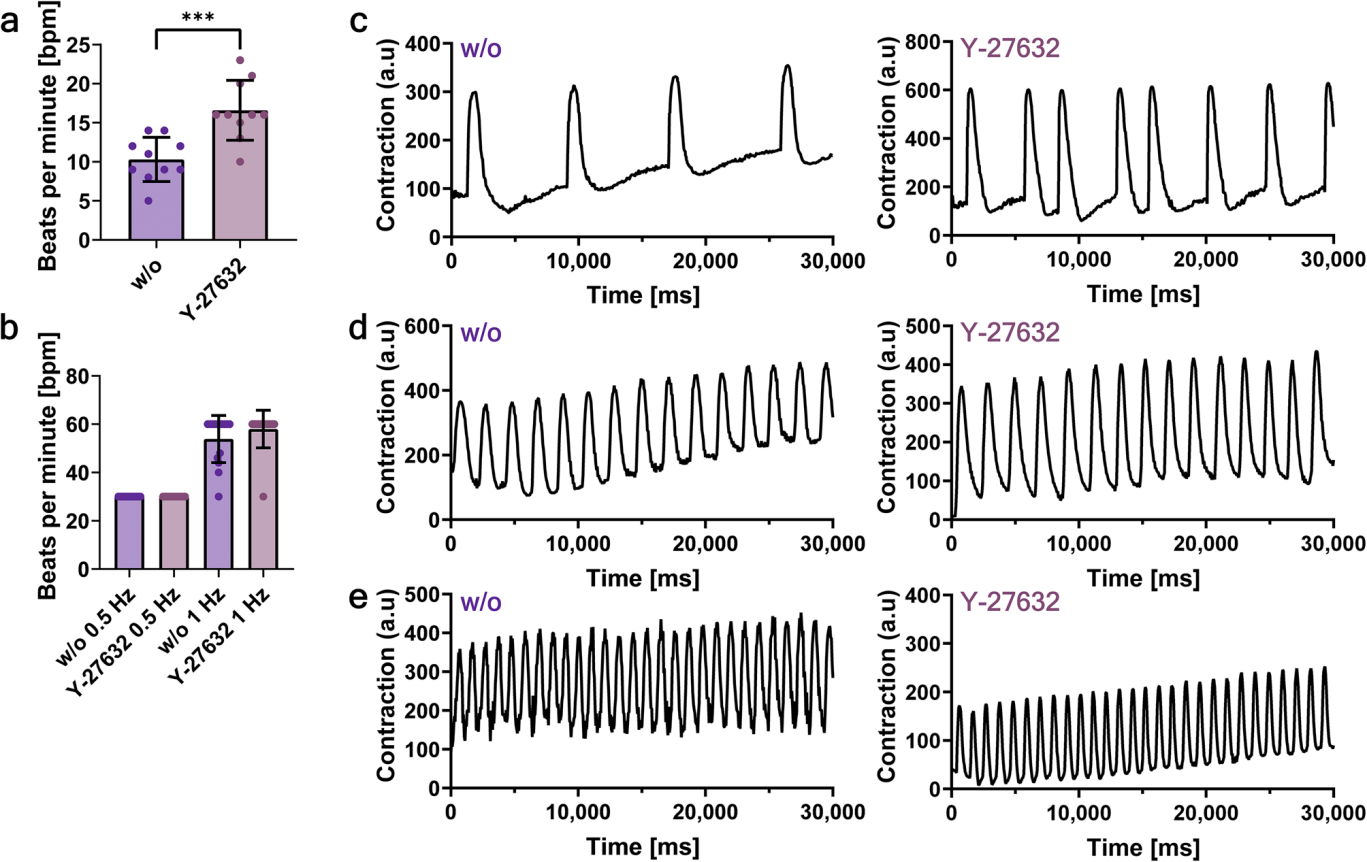
Functionality assessment of cryopreserved CMAs. After the 5d post-thaw culture, videos were taken for the subsequent analysis via the open-source software MUSCLEMOTION. (**a**) Mean distribution of the spontaneous contraction of CMAs post-thaw. Shown are individual values of aggregates from n = 3 experiments and mean values ± SD. Data were analyzed for statistical significance using an unpaired t-test (***p ≤ 0.001). (**b**) Mean distribution of electrically stimulated contraction at 0.5 Hz or 1 Hz of CMAs at d5 post-thaw. Shown are individual values of aggregates from n = 3 experiments. Representative contraction profile of (**c**) spontaneously contracting CMAs, (**d**) 0.5 Hz stimulated CMAs, or (**e**) 1 Hz stimulated CMAs at d5 post-thaw.

By d5 post-thaw the CMAs demonstrate spontaneous contraction (Fig. 5). However, CMAs cryopreserved without the Y-27632 pre-treatment had a significantly (***p ≤ 0.001) slower contraction with 10 bpm ± 3 bpm compared to CMAs cryopreserved with the Y-27632 pre-treatment which displayed a spontaneous contraction of 17 bpm ± 4 bpm (Extended Data Video 1 and 2 respectively). To further confirm whether these different spontaneous contraction rates were due to post-thaw stress, resulting in a negative impact on the overall function of cryopreserved CMAs, electric stimulation was performed. Age-matched fresh CMAs (dd9 and dd14) showed no spontaneous contraction and little to no induced beating upon electric stimulation (Extended Data Video 3). CMAs cryopreserved without or with Y-27632 pre-treatment could be paced at both 0.5 Hz and partially at 1 Hz (Fig. 5 and Extended Data Video 4–7).

## Discussion

The utilization of iPSC-derived cardiomyocytes for their clinical application is moving more into focus due to the ongoing optimization of established differentiation protocols toward scalable GMP compliant processes. The utilization of 3D-based methods is shifting into focus, as the cell–cell connectivity allows for application of the models for more in-depth analyses of tissue comparable to the organ, drug screening and cardiotoxicity assessment. However, a remaining bottleneck for the efficient use of cardiac cell-therapies is their safety and ready availability. Due to the time required for the GMP-compliant manufacturing of large quantities of cardiomyocytes, cryopreservation can be a useful tool for long-term storage to make cells easily available and to allow time for QA/QC assessments of the product for clinical use.

The objectives of this work were (1) to identify promising approaches for upscaling the optimized 4 factor-based cardiomyocyte differentiation protocol and (2) to investigate existing cryopreservation protocols in depth and optimize these, for the efficient freezing of complex cardiomyocyte aggregates.

In recent years the focus of cardiomyocyte differentiation has shifted from 2D monolayers^34,35^ to manufacturing in 3D-based suspension cultures^36^, due to the high cell numbers needed for the possible application of PSC-derived cardiomyocytes in a clinical setting. First scalable protocols demonstrate the production of highly pure (> 90% lineage positive cells, analyzed with the cardiac markers TNNT2, ACTN2 and MHC) CMAs on multiple platforms e.g., Erlenmeyer flasks (working volume 20 mL), DASbox Mini Bioreactor Systems (working volume 150 mL), stirred spinner flasks (working volume 300 mL) and stirred tank bioreactors (working volume 350-500 mL or 2000 mL)^13,14,37^. In contrast, the protocol utilized in this work established GMP iPSC lines with subsequent GMP-compatible workflows for differentiation into different cell types^12^. It was optimized for the production of CMAs in 6-well plates (working volumes 2-4 mL) with the manufacturing of > 95% pure CMAs (analyzed with the cardiac markers TNNT2, and ACTN2). First upscaling approaches utilizing Erlenmeyer flasks (working volume 20 mL) for the production of CMAs demonstrated the scalability and robustness of the protocol, as reflected by the consistently high cardiac marker expression and high viability of the cells independent of the platform. The results suggest that further up-scaling approaches to larger formats e.g., Erlenmeyer flasks with larger working volumes, or other bioreactors such as the vertical wheel, stirred-tank or the spinner flasks, should be achievable for simple production of GMP-compliant highly pure and viable CMAs. However, a remaining bottleneck in the production process and the subsequent utilization of PSC-derived cardiomyocytes in a clinical setting is the QA/QC assessments needed to ensure the safety of the product.

Most commonly, cryopreservation is utilized to enable banking of the cell product and allow time to evaluate all needed release criteria e.g., mycoplasma, sterility, and characterization of the product. Due to the new emergence of 3D-based differentiation models, most cryopreservation protocols available are tested mostly for the freezing of single cell cardiomyocytes that have been differentiated in 2D-monolayer cultures (Table 1). Unfortunately, most of the literature focuses on the efficiency of a cryopreservation procedure on analyses performed on d0 post-thaw. This leads to severe overestimation of the cryopreservation process as onsetting events such as apoptosis resulting from cryoinjury are not considered. Additionally, when only the viability of the cells is considered the number of cells lost during cryopreservation is not considered. However, for the application of cardiac cell-based therapies, a large number of viable cardiomyocytes will be needed, making cell recovery a crucial parameter that needs to be evaluated to determine the efficiency of the cryopreservation procedure.

**Table 1:** Overview of selected cardiomyocyte cryopreservation protocols listing data relevant to the assessments performed here.

References	Cell source	Freezing of	Cryopreservation medium	Selective results
Xu et al.^18^	hESC	Single Cells	Cryostor CS10	d0 Assessment: - Recovery of 70%—77% - Recovered spontaneous contraction after 1–2 days
Van den Brink et al.^19^	hiPSC	Single Cells	90% KOSR + 10% DMSO	d0 Assessment: - Viability via Trypan blue exclusion (> 90%) - Replating recovery (after 24 h) was only ~ 50% d3/d7 Assessment: - recovered contraction similar to fresh cells
Miller et al.^20^	hPSC	Single Cells	Comparison of: - Cryostor CS10 - 90% FBS + 10% DMSO - 90% KOSR + 10% DMSO - STEMdiff Cardiomyocyte Freezing Medium	d0 Assessment: - Recovery of 50%—70% d5/d7 Assessment: - Recovered spontaneous contraction - High TNNT2 marker expression
Maas et al.^21^	hiPSC	Single Cells	STEMdiff Cardiomyocyte Freezing Medium	d0 Assessment: - Viability via PI-Staining (~ 74%) - afterwards expansion with similar growth to fresh cells
Zhang et al.^22^	hiPSC	Single Cells	90% fetal bovine serum (FBS) + 10% DMSO	d0 Assessments: - Viability via Ca-AM and EtHD-1 (~ 60%) - Cardiac marker expression TNNT2 (> 90%)
Maas et al.^29^	hiPSC	Small Cardiomyocyte Spheroids	PSC Cryopreservation Medium (Thermofisher)	d0 Assessment: - Viability via PI-Staining (> 90%) d7 Assessment: - Viability via Ca-AM and EtHD-1 (~ 70%) - comparable cardiac marker expression for fresh vs. Cryopreserved cells (immunofluorescence staining) - recovery of beating (spontaneous contraction)
Prondzynski et al.^23^	hiPSC	Single Cells	STEMdiff Cardiomyocyte Freezing Medium	d0 Assessment: - Viability via Trypan blue exclusion (> 90%) d1 Assessment: - Plating efficiency (~ 50%)

We propose a panel of different QA/QC assessments to determine the efficiency of the cryopreservation procedure for CMAs. A short post-thaw culture period is essential for evaluating the extent of cryoinjury in cells and the subsequent onset of apoptosis. Here, 5 days of post-thaw culture is sufficient to allow time for delayed cryoinjury to take effect and assess the recovery of the CMAs. Immediately after thawing the viability of whole aggregates was assessed to determine the overall state of the CMAs and evaluate the core of the aggregates for cell death, as the overall size and density can hinder the ability of the cryoprotectant to penetrate fully into the CMA and therefore diminish the cryoprotective effect. The high viability on d0 post-thaw was consistent with findings from other groups (Table 1^18–23,29^). Additionally, to analyze cryoinjury during/after cryopreservation, which is often associated with apoptotic activity^17,38^, an apoptosis assay on d0 post-thaw was utilized to measure the extent of cryoinjury and account for possible low cell recovery rates both immediately post-thaw and after 5 days of post-thaw culture. This confirmed the onset of early apoptosis in cryopreserved CMAs and highlighted the need for a post-thaw culture, allowing the progression of apoptosis to not overestimate any of the tested freezing conditions. The loss of viable cells was further confirmed by cell recovery measurements on d5 post-thaw, which is heavily overlooked in other existing studies. Another important QA/QC assessment was the unchanging phenotype of the cells. Here, we focused on assessing the cardiac markers TNNT2 and ACTN2 on d5 post-thaw to ensure the analysis of recovered and viable CMAs. However, as there was no measured phenotypic drift of the cells, cardiac marker analysis can be done either on d0 post-thaw^22^ or d5 post-thaw^20,29^, as the literature suggests that marker expression is not affected by cryopreservation. Finally, we proposed a potency QA/QC assessment of the cells by analyzing the spontaneous contraction of the CMAs and their reaction to electric stimuli. As described above and as stated in other studies^18,19,23,29^, the cryopreservation procedure had no adverse effect on the function of cardiomyocytes, CMAs started to show signs of spontaneous contraction and were reactive to external electric stimulation. Cryopreservation may be a useful tool to push CMAs towards structural maturation, as the fresh CMAs with the more cystic morphology did not show spontaneous contraction and little to no reaction to electric stimulation. The cryopreserved CMAs had a more condensed morphology after the 5-day post-thaw culture and showed functional properties.

A remaining problem in CMA cryopreservation is batch-to-batch variability when comparing cell recovery rates. The size of the aggregates made accurate counting of freshly differentiated CMAs (dd9) difficult, resulting in possible over- or underestimation of the number of cells, highlighting the need for better standardization of cell counting of larger tissues like CMAs. A possible proposal to circumvent this problem is the investigation of a dissociation and subsequent re-aggregation process of CMAs. Preliminary data shows a more homogenous formation of smaller and denser CMAs.

Although the aggregate structure prior to cryopreservation is quite robust, any excessive disruption of the CMAs during the pipetting steps should be prevented by a gentle handling. Additionally, as the morphology of cryopreserved CMAs suggests, the aggregate structure was very fragile post-thaw. Additional shear stress from the washing steps and the dissociation process could have resulted in cell loss and consequently low recovery rates.

Further optimization of the cryopreservation process was attempted by adding a 1 h pre-incubation with 10 µM Y-27632 to inhibit apoptosis triggered by freezing. Previous studies have suggested that pre-treatment of cardiomyocytes before cryopreservation improves the overall number of surviving cells as well as their attachment and beating recovery^39^. However, the findings of the present study did not confirm an improvement in overall cell recovery with Y-27632 pre-treatment. This could be due to the fact that various mechanisms result in cell death after cryopreservation, which may make it necessary to inhibit different pathways of cellular stress responses for advanced cryoprotection^40^.

**Fig. 6:**
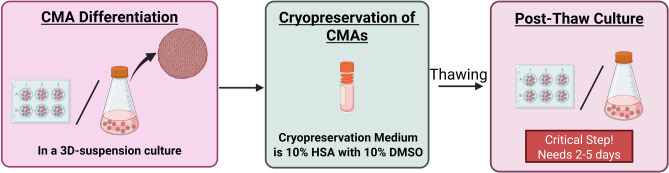
Schematic of the most promising cryopreservation medium selected for the freezing of CMAs. Created in https://BioRender.com.

In conclusion, the results presented here show the robust scalability of the 4-factor based cardiomyocyte differentiation protocol for the efficient production of highly pure and viable CMAs. Effective cryopreservation of CMAs has been achieved with high recoveries, non-compromised phenotype, viability, or function after a short post-thaw culture (d5), with the optimization of a cost-effective and GMP-compliant process utilizing “10% HSA” as the cryopreservation medium of choice (Fig. 6). This protocol will be further optimized to combat high batch-to-batch variability and ensure translation of the process for other cell lines.

For a more comprehensive understanding of the effect of cryopreservation of CMA freezing different methods such as morphological assessment, apoptosis analysis, recovery calculation immediately post-thaw and after an appropriate post-thaw culture time, cardiac marker expression analysis, and functional assessment, were established.

The protocol may be utilized for the project HEAL (funding number 101056712), which aims to administer cardiomyocyte aggregates in a pre-clinical trial, as previous animal studies have shown a better engraftment of CMAs in the heart as compared to their single cell counterparts^41,42^.

## Experimental methods

### Cardiomyocyte differentiation from hiPSC in 3D suspension culture

Human cord blood CD34^+^ hematopoietic stem cells (HSC) were isolated and short term expanded from selected HLA-homozygous (HLAh) cord blood donations, after the donors re-consent, as previously described^43^. The GMP-grade reprogramming for the generation of clinical-grade HLAh-iPSC lines was performed as described elsewhere^12^. Briefly, episomal reprogramming was based on the protocol of Okita et al.^44^, after which the iPSC lines underwent QA/QC testing including sterility and mycoplasma testing, endotoxin level and viral contamination screening, and genomic integrity analysis. The reprogrammed lines were tested for their differentiation capability into retinal pigment epithelium (RPE) cells, mesenchymal stromal cells (MSC), cardiomyocytes, hematopoietic stem cells (HSC), T- and NK-cells as well as monocytes.

The iPSC line used in this study (R26) was provided in accordance with the participation in the synergistic consortium funded by EU HORIZON-HLTH-2021-TOOL-06–02 (101056712) and has the HLAh haplotype A*01:01; B*08:01; C*07:01; DRB1*03:01; DQB1*02:01.

The iPSC maintenance and cardiomyocyte differentiation was based on the optimized protocol established by Terheyden-Keighley et al.^12^. In brief, iPSCs were cultured in StemMACSiPS-Brew XF medium (Miltenyi Biotec, Product No. 130–104-368) on 0.16 µg/cm^2^ iMatrix-511 (Amsbio, Product No. AMS.892 011); splitting was performed twice a week using Accutase (Merck, Product No. A6964) with the addition of 10 µM Y-27632 (R&D Systems, Product No. 1254).

Cardiomyocyte differentiation was carried out in 3D suspension culture, using either 6-well plates (Corning, Product No. 3516) or 125 mL Erlenmeyer flasks (VWR, Product No. 214–0447) placed on a wave shaker (65 rpm) or an orbital shaker (80 rpm), respectively. In modulation of the established protocol, cardiac differentiation was initiated with 0.5*10^6^ iPSC/mL to 0.75*10^6^ iPSC/mL. In brief, the basic cardiomyocyte differentiation medium (basic-CM) consisted of Knockout DMEM (Thermofisher, Product No. 10829018), 250 µM 2-phospho-L-ascorbate (Sigma-Aldrich, Product No. 49752), 0.05% (w/v) HSA (Octapharma, albunorm 20%), and 2 mM L-glutamine (Thermofisher, Product No. 25030081). Differentiation was started by adding basic-CM medium to single cell iPSC, with the addition of 1:1000 ITS (Fisherscientific, Product No. 10102281), 10 µM Y-27632, 0.5 µM CHIR99021 (Axon Medchem, Product No. 1386), 5 ng/mL BMP-4 (R&D Systems, Product No. 314-BP), 7.5 ng/mL FGF-2 (Peprotech, Product No. 100-18B), and 7.5 ng/mL Activin-A (R&D Systems, Product No. 338-AC). On dd2, 25% of the culture medium was replaced with basic-CM medium with the addition of 2 µM C-59 (Tocris, Product No. 5148) (0.5 µM final concentration in the culture). On dd4 and dd7, the medium was replaced with basic-CM medium without any further additives. From dd9 onward, cells were assessed for their cardiomyocyte content and used for downstream analyzes.

All cells were cultured in a cell culture incubator at 37 °C with 5% CO_2_.

### Cryopreservation of cardiomyocyte-aggregates

CMAs from dd9-dd11 were utilized for cryopreservation. The medium was changed to fresh basic-CM medium 24 h prior to freezing. On the day of freezing, cells were cooled down to 4 °C in the fridge for 0.5 h. Cell numbers per cryovial (Greiner Bio-one, Product No. 122263) ranged from 1*10^6^ to 4*10^6^. After centrifugation (200 xg, 5 min, 4 °C), the supernatant was discarded, and the cells resuspended in the respective freezing medium (Table 2). While adding the respective freezing medium cells were always kept on a cooling pack to maintain a temperature of ~ 4 °C. Cryovials were transferred into a CoolCell™ Freezer Container (Corning, Product No. 432138) and frozen in a -80 °C freezer overnight, after which they were put into liquid nitrogen (-196 °C) for long-term storage.

**Table 2:** Composition of the utilized freezing media.

Freezing medium	Company and product number	Volume for freezing	Label
STEMdiff™ Cardiomyocyte Freezing Medium	Stemcell Technologies, Product No. 05030	1 mL	Stemdiff-CMFM
CryoStor® CS10	Stemcell Technologies, Product No. 07959	1 mL	CS-10
NutriFreez® D10 Cryopreservation Medium	Sartorius, Product No. 05–713-1	1 mL	Nutrifreeze-D10
STEM-CELLBANKER – GMP Grade	Amsbio, Product No. 11897	1 mL	Stem Cellbanker
basic-CM medium with the addition of HSA (20%) and CryoSure	Not applicable	1.333 mL	10% HSA
basic-CM medium with the addition of 10% KnockOut™ Serum replacement and CryoSure	Not applicable	1.333 mL	10% KOSR
90% KnockOut™ Serum replacement and CryoSure	Not applicable	1.333 mL	90% KOSR
basic-CM medium and CryoSure	Not applicable	1.333 mL	CMFreeze

For the optimization of the cryopreservation procedure, two different parameters were investigated:

1. Comparison of different freezing media.

For the commercially available media, the cell preparation prior to cryopreservation was performed as recommended by the respective company. The freezing media containing CryoSure (WAK-Chemie, Product No. WAK-DEX40-NaCL-10) were prepared to contain 10% DMSO in the final volume.

2. Effect of Y-27632 on cryopreservation.

CMAs were incubated with 10 µM Y-27632 in the 3D suspension culture at 37°C 1h prior to cryopreservation.

### Thawing of cardiomyocytes

Cryovials were removed from liquid nitrogen and thawed in a 37 °C water bath. For uniform thawing, the vial was gently moved in the water bath until only a small ice clump was left, after which the content of the vial was transferred into a 50 mL tube. For a gentle thawing, 1 mL pre-cooled 5% HSA solution was used to recover any cells left in the cryovial. The suspension was then transferred dropwise into the 50 mL tube containing the thawed cells, over 1 min under gentle swirling of the tube. Another 1 mL thawing solution was added dropwise in the same manner. After letting the cells equilibrate for 30 s to 1 min, 8 mL thawing solution was added dropwise to the tube over 1 min. The cells were washed twice with PBS by centrifuging at 300 xg for 4 min at 4 °C and discarding the supernatant without disrupting the cell pellet. Afterwards the cells were transferred to suspension culture on a shaker in basic-CM medium with the addition of 10% KnockOut™ Serum replacement (Thermofisher, Product No. 10828010) and 10 µM Y-27632 for the first 48 h. On d2 post-thaw, 50% of the post-thaw culture medium was discarded and replaced with double the amount of fresh basic-CM medium.

For steps requiring the aggregates to be transferred with a 1000p pipette, it is recommended to use cut tips or tips with a bigger lumen, to ensure as little disruption of the aggregates via shear force as possible.

### Morphology assessment and size measurement

For the morphological assessment of cells during differentiation and post-thaw, bright field images were taken with an Olympus microscope (CKX41) and a mounted CC-12 Soft Imaging System utilizing the software cell^D (Version 2.7) for the image acquisition. For the manual measurement of the aggregate diameter, ImageJ ((Fiji Is Just) ImageJ Version 2.14.0) was utilized.

### Dissociation of cardiomyocyte aggregates

A sample of CMAs was collected and washed once in PBS (centrifugation 300 xg for 3 min). After discarding the supernatant, the cell pellet was resuspended in 500 µL pre-warmed STEMdiff™ Cardiomyocyte Dissociation Medium (Stemcell Technologies, Product No. 05026) and incubated for 10 min at 37 °C. At the end of the incubation, the cells were resuspended up to 10 times with a 1000p pipette to ensure aggregate breakage and afterwards the dissociation process stopped by adding 500 µL of basic-CM medium. After centrifugation (600 xg for 5 min), supernatant was discarded, and the cell pellet resuspended in PBS (no more than 10 times of resuspension with a 1000p pipette). Single cell CMs were counted via trypan blue staining and manual counting utilizing a Neubauer counting chamber.

### Flow cytometry

After dissociation, single cell CMs were stained with BD Horizon™ Fixable Viability Stain 780 (BD Pharming, Product No. 565388) according to the manufacturer’s protocol. In short, after preparation of the final aliquots from the stock solution, up to 1*10^6^ were stained 1:100 in PBS for 15 min at RT in the dark. Afterwards, samples were washed with PBS (centrifugation at 500 xg, 3 min). For intracellular staining the Transcription Factor Staining Buffer Set (Miltenyi Biotec, Product No. 130–122-981) was utilized according to the manufacturers protocol. In short, cells were fixed and permeabilized with Solution 1 and Solution 2 at a working concentration of 1:4, for 30 min at RT. After centrifugation (500 xg for 3 min), the supernatant was discarded, and the cells were resuspended in 1 × permeabilization buffer and incubated for 30 min at 4 °C in the dark. During the permeabilization step, staining with an isotope control IgG (Miltenyi Biotec, Product No. FITC 130–113-449, PE 130–113-450), cardiac markers ACTN2 (Miltenyi Biotec, Product No. 130–119-766), or TNNT2 (Miltenyi Biotec, Product No. 130–119-575), iPSC pluripotency marker POU5F1 (Miltenyi Biotec, Product No. 130–131-113) or proliferation marker MKI67 (Miltenyi Biotec, Product No. 130–120-417) was done at a dilution of 1:100. Cells were analyzed via flow cytometry (Cytoflex, Beckman Coulter).

### Cell cycle analysis

The cell cycle analysis was based on the protocol published by Riccardi and Nicoletti^45,46^. In short, single cell cardiomyocytes were resuspended in a fluorochrome solution consisting of 0.1% (w/v) sodium citrate, 0.1% (v/v) Triton X-100, and 50 mg/L Propidium-Iodide in distilled water. The flurochrome solution was added 1:1 to the cell suspension. After direct resuspension, the samples were stained for 5–10 min, after which they were again resuspended and then assessed via flow cytometry. This analysis allowed for differentiation between the different cell cycle phases: sub-G_1_, G_0_/G_1_, S, and G_2_/M.

### Apoptosis assay

For a more precise apoptosis analysis, FITC Annexin V Apoptosis Detection Kit I (BD Pharming, Product No. 556547) was utilized according to the manufacturer’s protocol. In short, after the dissociation of CMAs, the single cells were resuspended in 1 × Annexin V Binding Buffer and incubated with Annexin V and PI for 15 min at RT in the dark. After incubation, cells were analyzed via flow cytometry within 1 h of staining (CytoFlex, Beckman Coulter).

### Total RNA extraction, reverse transcription (RT) and real-time quantitative polymerase chain reaction (qPCR)

Total RNA was extracted from cells utilizing the RNeasy Kit (Qiagen, Product No. 74104) according to the manufacturer’s instruction. The concentration and quality of RNA was determined using a Nanodrop (Thermofisher, NanoDrop ND-1000 Spectrophotometer). The RT was carried out with the first-strand cDNA synthesis kit (Thermofisher, Product No. K1612) utilizing the oligo(dT)_20_ primer according to the manufacturer’s instruction. The qPCR was performed utilizing SybrGreen-Mix (Thermofisher, Product No. AB1322B) and gene specific primers (Thermofisher, Custom Standard DNA Oligos; miR-302/367 HT Forward primer sequence TGGAGGAGAACACGAATCTTTGG / Reverse primer sequence GAACAGGGAAGAGGAAGAGAAGCA) with 50 ng of cDNA. The real-time qPCR was run on a Quantstudio3 (40 Cycles: 15 s 95 °C, 1 min 60 °C) and analyzed utilizing the QuantStudio™ Design and Analysis Software (Version v1.5.2). Relative gene expression changes were calculated with the comparative ΔΔCT method, normalizing CT values to the housekeeping gene RPL13a.

### Fluorescence viability staining

Whole aggregate/spheroid viability of both fresh and thawed CMAs was analyzed via a live/dead staining as described by Nils Kriedemann et al.^13^. A 2 mM stock solution of calcein AM (Ca-AM) (Sigma Aldrich, Product No. 17783) and ethidium homohimer-1 (EtHd-1) (Sigma Aldrich, Product No. 46043) was prepared with DMSO. For a fresh working solution, 0.4 µM Ca-AM and 0.8 µM EtHd-1 were added to PBS and stored in the dark until use. After washing a sample of CMAs with PBS (300 xg for 3 min), cells were gently resuspended in 2 mL working solution and transferred into a 6 well plate. For the staining, the cells were incubated for 30 min to 1 h at 37 °C in the dark on a shaker. A fluorescence microscope (Axio Observer with a mounted Axiocam 712) and the Zen software (Version 3.1) were used for the acquisition of the Ca-AM signal (ex/em 494/517 nm) and the EtHd-1 signal (ex/em 528/ 617 nm).

### Functionality assessment – spontaneous contraction

Spontaneous contraction of CMAs was recorded as bright field or phase-contrast movies utilizing an Axiocam 503 mono camera mounted on a Zeiss Axio Vert.A1 microscope utilizing the Zen Software (Version 3.1). Video analysis was performed via the open-source software MUSCLEMOTION, as an ImageJ plug-in, in accordance with the publisher^47,48^.

### Functionality assessment – electric stimulation

To analyze electric stimulation of CMAs, the cells were washed ones with PBS and put into a freshly prepared buffer (pH 7.4) consisting of 137 mM NaCl (Merck, Product No. 1.06404.1000), 5.4 mM KCl (Roth, Product No. HN02.1), MgCl_2_ × 6H_2_0 (Sigma Aldrich, Product No. M2393), 10 mM HEPES (Roth, Product No. HN77.4), and 1.2 mM CaCl_2_ (Honeywell, Product No. 15641930). Cells were imaged using a Zeiss Axiovert S100TV with a mounted Basler camera (acA640-750um) and the ImageJ plug-in Micro-Manager Reader (Version 1.3.38). For the electric stimulation, electrodes were put directly into the buffer containing the aggregates and a voltage of 10 mA was applied with the frequency of either 0.5 Hz or 1 Hz with the A-M Systems Isolated Pulse Stimulator Model 2100. Analyzes were performed as described in the previous section.

### Statistical analysis

Data was analyzed and graphed in GraphPad Prism (Version 8.0.2). Where applicable, data was presented as mean ± SD. Comparisons were calculated utilizing unpaired t test, Welch’s correction was applied if the compared data had unequal variances; significance was defined by *p ≤ 0.05, **p ≤ 0.01, ***p ≤ 0.001, ****p ≤ 0.0001.

## Supplementary Information

Supplementary Information 1.

Supplementary Video 1.

Supplementary Video 2.

Supplementary Video 3.

Supplementary Video 4.

Supplementary Video 5.

Supplementary Information 2.

## Data Availability

The original data supporting the findings of this study are available within the article and in the supplementary material. Further inquiries for raw data files can be directed to the corresponding author upon reasonable request.
