# Supplementary material for: Improved cryopreservation of cardiomyocyte aggregates differentiated from GMP iPSC in a 3D culture format
Source: Sci Rep. 2026 Jan 12;16:1640. doi: 10.1038/s41598-025-32439-3 (PMC12800097; doi:10.1038/s41598-025-32439-3)
Supplement: Supplementary file 1 — Supplementary Information 1. [file 41598_2025_32439_MOESM1_ESM.docx]

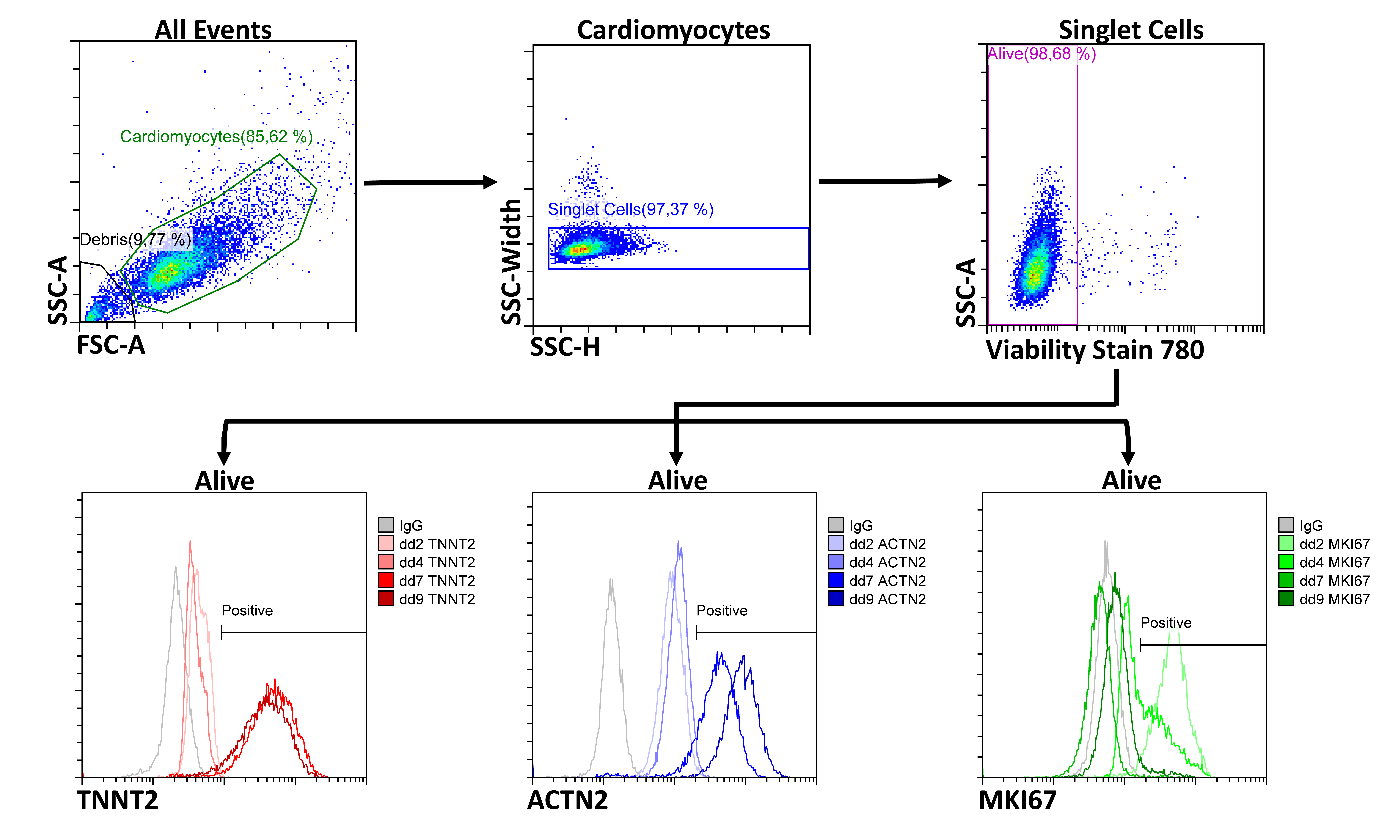


**Extended Data Fig. 1: Flow Cytometry Successive Gating for the Characterization of Cardiomyocytes.** SSC-A vs. FSC-A allows for the exclusion of debris and the selection of the target cell population; SSC-Width vs. SSC-H to select singlet cells; the Viability Stain 780 was utilized to determine alive cells; Based on the alive cell population TNNT2^+^, ACTN2^+^, or MKI67^+^ populations were determined.


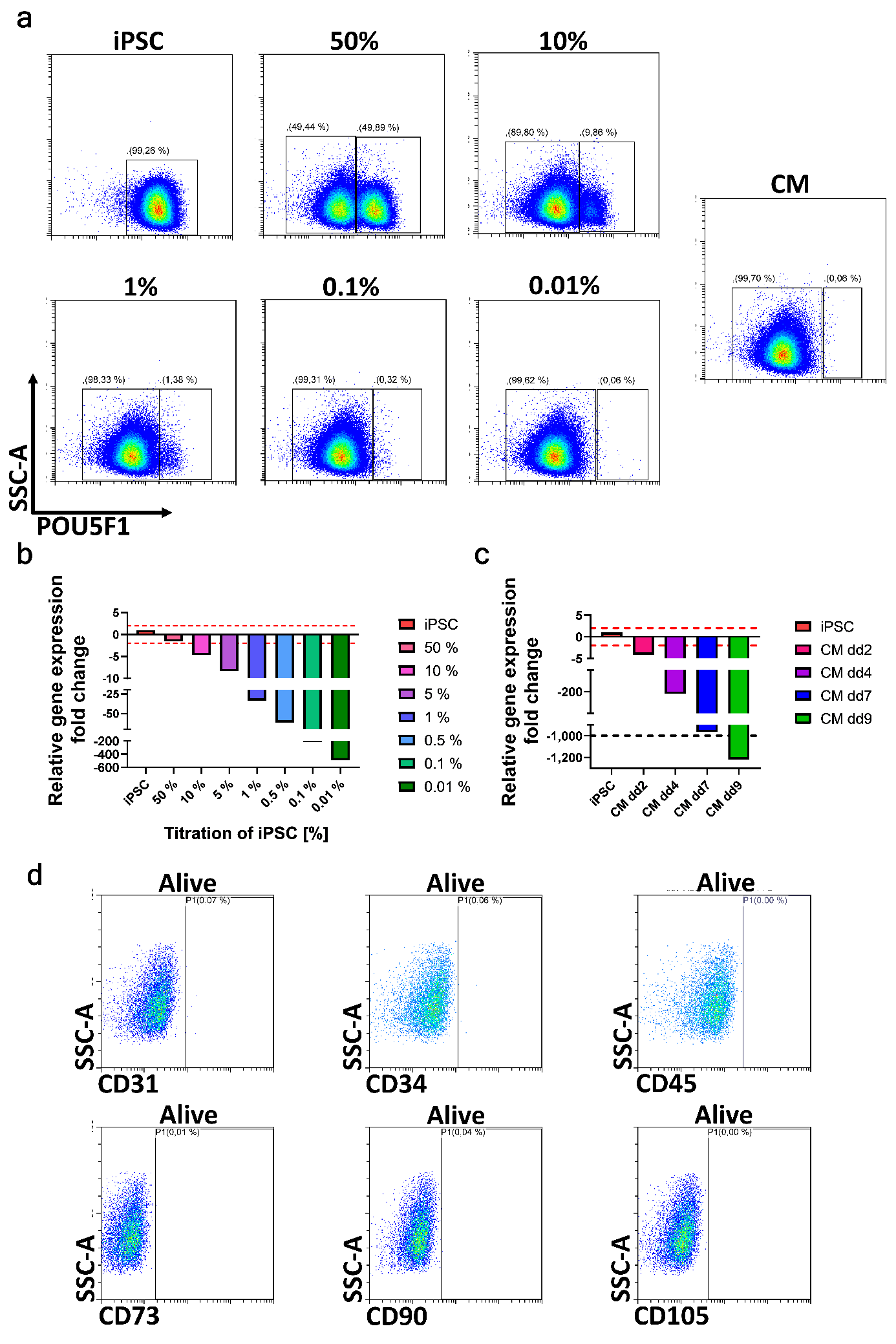


**Extended Data Fig. 2: Quality control assessment for residual contaminating iPS cells and non-CM populations.** (a) Flow cytometry detection of POU5F1 with different concentrations of iPSC (50 % to 0.01 %); (b) Detection limit determination for the expression of miR302/367 HT with different concentrations of iPSC (50 % to 0.01 %) via qPCR; (c) Determination of residual iPSC during the 9-day differentiation of cardiomyocytes, the threshold of -1000-fold was determined as the value for no residual iPSC present in the cell sample; the red dotted lines at 2-fold and -2-fold mark significant up- or down-regulation of the gene expression; (d) Based on the alive cell population in flow cytometry selected markers were utilized for a broad overview of possible lineages that could have developed from the differentiation (representative plots for n=6 replicates).


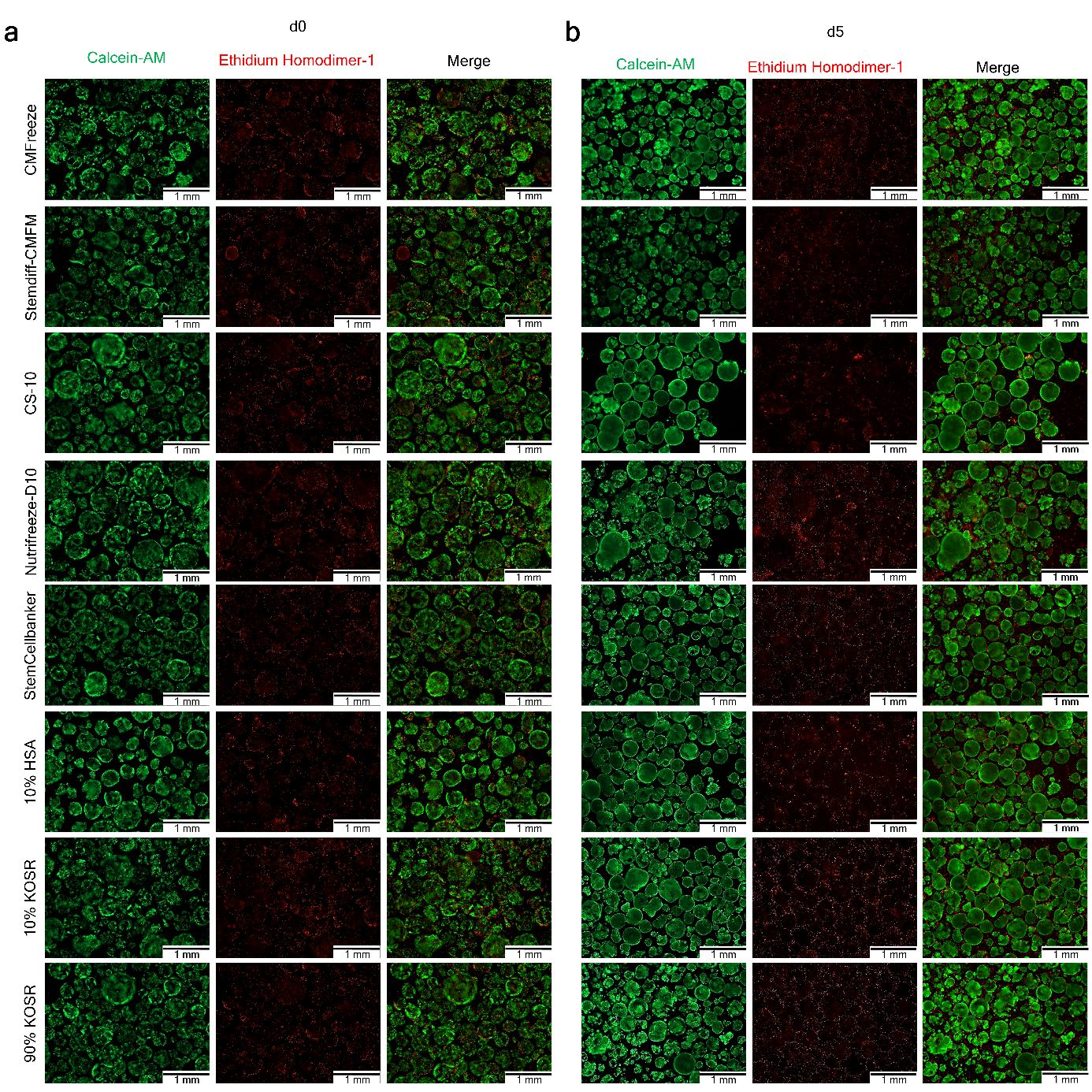


**Extended Data Fig. 3: Viability assessment of cryopreserved whole aggregates**. Representative fluorescent images of CMAs d0 (a) and d5 (b) post-thaw for viability assessment. Staining with Calcein-AM (green) and Ethidium homodimer-1 (red). Scale bar 1 mm.


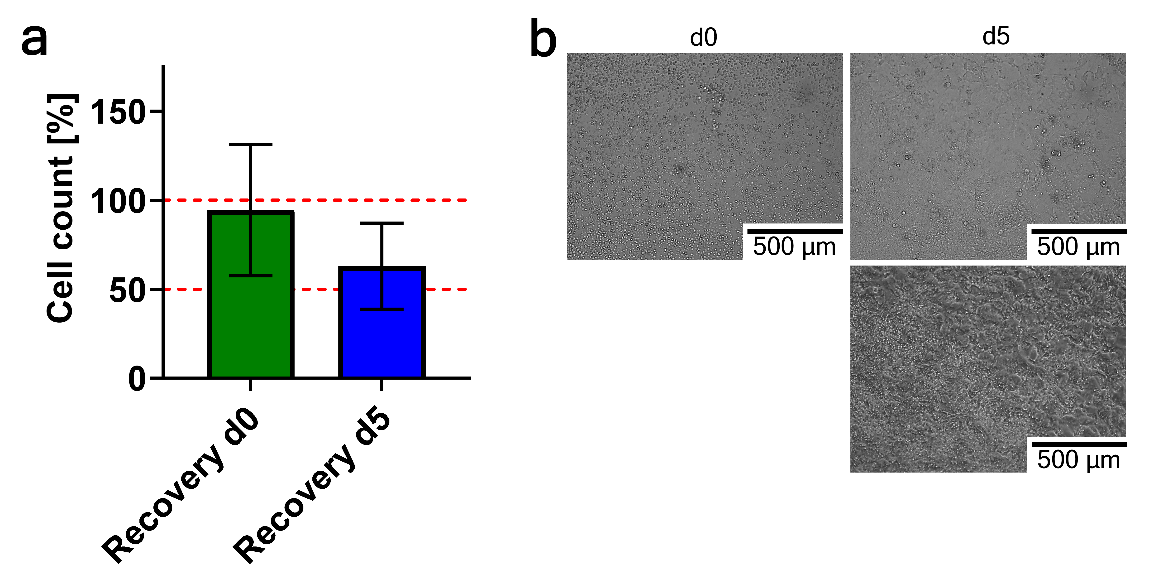


**Extended Data Fig. 4: Cryopreservation of Single Cell Cardiomyocytes with 10% HSA.** (a) Recovery at d0 post-thaw and after a 5-day post-thaw monolayer culture (mean values ± SD, n=3). (b) representative morphological images of the monolayer cardiomyocyte culture.


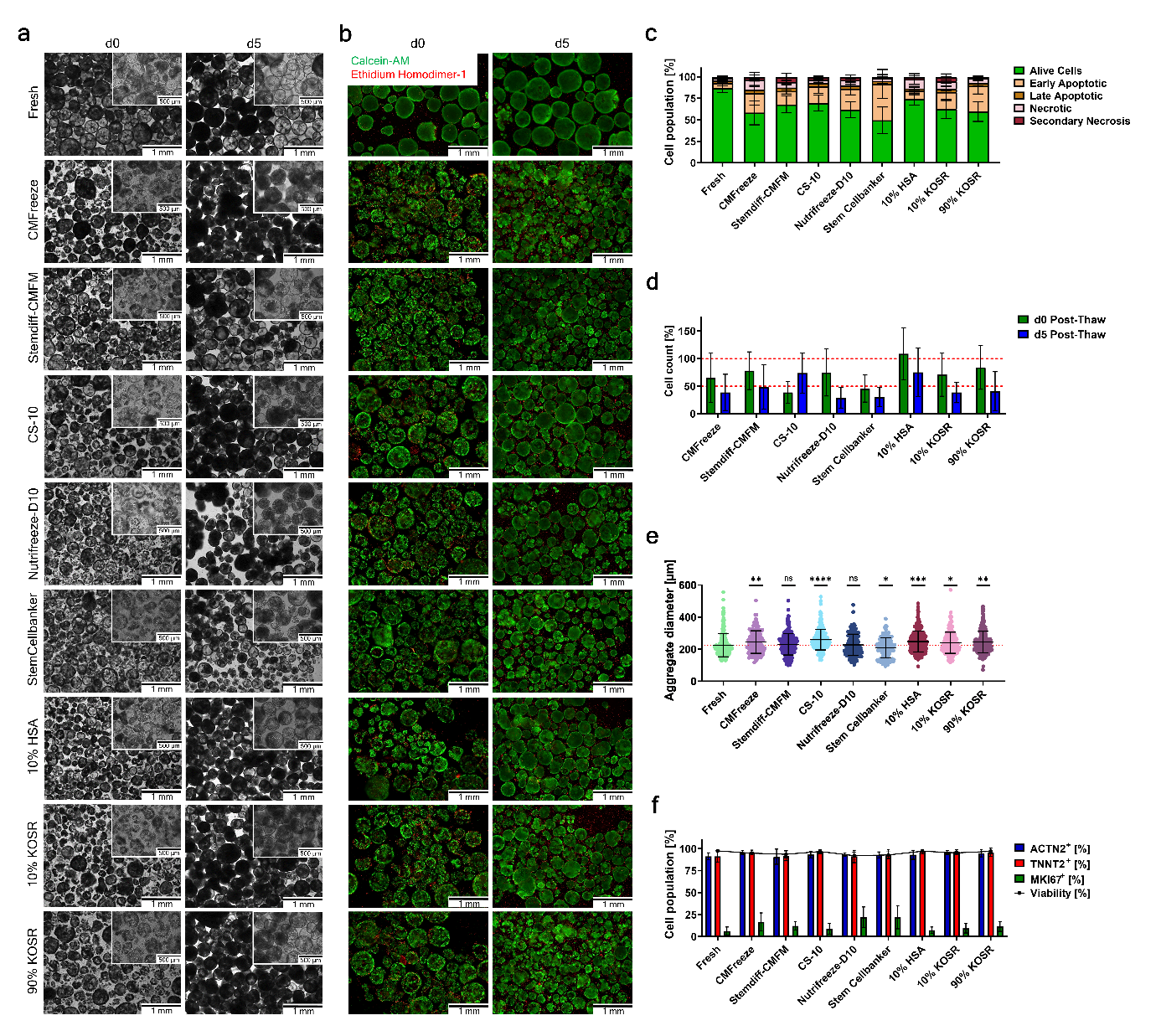


**Extended Data Fig. 5: Cryopreservation of Cardiomyocyte-Aggregates pre-treated with Y-27632.** Comparison of different freezing media for the cryopreservation of pretreated CMAs. (a) Representative bright field images for morphological assessment of CMA integrity d0 and d5 post-thaw. Scale bar 1 mm and 500 µm. (b) Representative fluorescent images of CMAs d0 and d5 post-thaw for viability assessment. Staining with Calcein-AM (green) and Ethidium homodimer-1 (red). Scale bar 1 mm. (c) Apoptosis assay via annexin V / PI on d0 post-thaw. (d) Cell recovery analyzed immediately on d0 post-thaw and after a 5-day post-thaw culture (n = 5-8, mean values ± SD, the red dotted line marks both 50 % and 100 % recovery)). (e) Aggregate diameter distribution on d5 post-thaw; shown are individual values of aggregates from n=5-8 experiments and mean values ± SD (for each experiment >30 aggregates were measured, the red dotted line indicated the mean value of the fresh sample); Data were analyzed for statistical significance using an unpaired t-test (*p ≤ 0.05, **p ≤ 0.01, ***p ≤ 0.001, ****p ≤ 0.0001). (f) Flow cytometry analysis for cardiac markers ACTN2 and TNNT2, proliferation marker MKI67, and viability on d5 post-thaw (mean values ± SD, n=5-8). In all analyzes the condition “Fresh” indicated CMAs analyzed prior to freezing as an age matched alive control; n=5 referred to the conditions CMFreeze, Nutrifreeze-D10, and Stem Cellbanker; n=8 referred to Stemdiff-CMFM, CS-10, 10% HSA, 10% KOSR, and 90% KOSR.


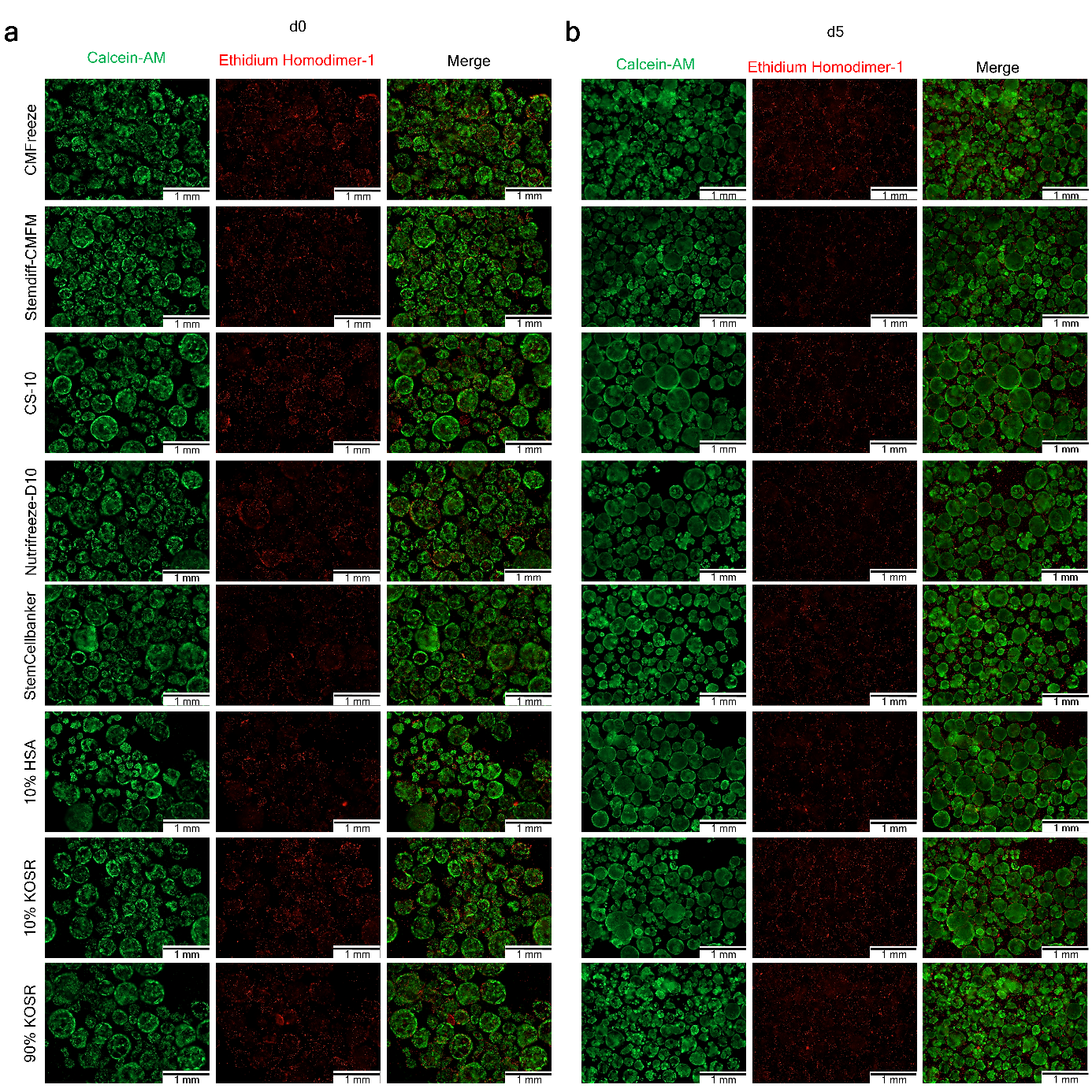


**Extended Data Fig. 6: Viability assessment of cryopreserved whole aggregates pre-treated with Y-27632**. Representative fluorescent images of CMAs d0 (a) and d5 (b) post-thaw for viability assessment. Staining with Calcein-AM (green) and Ethidium homodimer-1 (red). Scale bar 1 mm.
