# Supplementary material for: Improved cryopreservation of cardiomyocyte aggregates differentiated from GMP iPSC in a 3D culture format
Source: Sci Rep. 2026 Jan 12;16:1640. doi: 10.1038/s41598-025-32439-3 (PMC12800097; doi:10.1038/s41598-025-32439-3)
Supplement: Supplementary file 7 — Supplementary Information 2. [file 41598_2025_32439_MOESM7_ESM.docx]

**Supplement Extended Data Video Legends**

**Supplement Extended Data Video 1:** d5 spontaneous contraction of with 10% HSA cryopreserved CMAs without pre-treatment.

**Supplement Extended Data Video 2:** d5 spontaneous contraction of with 10% HSA cryopreserved CMAs with Y-27632 pre-treatment.

**Supplement Extended Data Video 3:** Electrical Stimulation of fresh CMAs at dd14

**Supplement Extended Data Video 4:** Electrical Stimulation (0.5 Hz) of with 10% HSA cryopreserved CMAs without pre-treatment.

**Supplement Extended Data Video 5:** Electrical Stimulation (1 Hz) of with 10% HSA cryopreserved CMAs without pre-treatment.

**Supplement Extended Data Video 6:** Electrical Stimulation (0.5 Hz) of with 10% HSA cryopreserved CMAs with Y-27632 pre-treatment.

**Supplement Extended Data Video 7:** Electrical Stimulation (1 Hz) of with 10% HSA cryopreserved CMAs with Y-27632 pre-treatment.
